# Perspectives on Vaginal Ecology and Management of Recurrent Vulvovaginal Candidiasis: A Narrative Review

**DOI:** 10.3390/jof11110806

**Published:** 2025-11-13

**Authors:** Danilla Grando, Cathy J. Watson

**Affiliations:** 1 School of Science, RMIT University, Melbourne, VIC 3000, Australia; 2 Centre for Epidemiology and Biostatistics, Melbourne School of Population and Global Health, The University of Melbourne, Carlton, VIC 3053, Australia; cathyw@unimelb.edu.au; 3 Women’s Health Clinics, The Royal Women’s Hospital, Parkville, VIC 3052, Australia

**Keywords:** vulvovaginal candidiasis, vaginal microbiome, management of recurrent vulvovaginal candidiasis, community state types

## Abstract

Symptomatic vulvovaginal candidiasis (VVC) affects around three-quarters of women at least once in their lifetime. Around 10% of these women will experience prolonged or recurrent vulvovaginal candidiasis (RVVC), which fails to respond, despite following recommended therapy. Most commonly prescribed therapy involves suppression therapy—usually for two weeks—which aims at eliminating symptoms by frequent administration of antifungals, followed by maintenance (weekly/monthly) therapy for up to six months. However, following cessation of maintenance therapy, around 50% of these women experience relapse. The vaginal ecology of RVVC can be characterized, and it is thought that biofilms and/or the development of antifungal resistance prevent adequate resolution. However, hypersensitivity may also confound management. This narrative review was performed to identify key studies that examine the management of VVC and the challenges of current prolonged antifungal therapy. It identifies gaps that show it remains important to investigate microbiological findings in RVVC and how these may inform rational choices in therapy in an era of rising antimicrobial resistance. Hope exists, as studies of the vaginal microbiome highlight that the type of microbiota may influence the level of inflammation and reduce symptomatology. Future research will continue to explore whether a personalized medicine approach can promote healthy vaginal ecology and prevent the debilitating long-term effects of RVVC.

## 1. Introduction

Vulvovaginal candidiasis (VVC) is estimated to affect at least 70–75% of females sometime during their reproductive life [1] and is uncommon in post-menopausal women unless exogenous estrogen is prescribed [2]. Colonization of the genital area with Candida may be asymptomatic, but some women can experience clinical manifestations including itching, particularly in the vulva area; a curd-like discharge; and stinging or burning on micturition. Oral antibiotics, diabetes, use of hormone therapy and pregnancy have been associated with genital candidiasis in women. Treatment is often initiated by the patient, as there are topical and oral anti-fungal medications available without prescription in Australia. For most women, symptoms resolve spontaneously or with anti-fungal treatment.

However, women may present to general practitioners (GPs) when symptoms are prolonged or recurrent. For some women, this may entail several visits to the GP and failure to eradicate symptoms despite using recommended therapy, which involves ‘suppressive and maintenance therapy’, usually for up to six months [3]. Using this therapy, at least 50% of women may experience recurrences once treatment finishes. With failure of mainstream management, the impact on women’s lives can be significant [4,5], and many women seek alternative methods of management [6], which may be independent from—or as an adjunct to—mainstream management [7]. The issue then becomes the following: How do we manage cases of recurrent vulvovaginal candidiasis (RVVC), which can have wide-ranging impacts on the lives of sufferers?

Some sources define RVVC as three symptomatic episodes of VVC in 12 months—others, four [3,8]. This may be because of changing expert consensus [8] or some definitions including the necessity of mycological evidence in defining cases, or it may reflect the scarcity of unbiased sampling worldwide, as well as the inaccuracy of self-diagnosis [9]. For the purpose of this review, RVVC was arbitrarily defined as four or more episodes of vulvovaginal candidiasis per year, as has been reported in up to 9% of sufferers [9,10]. Based on this percentage, Willems et al. [11] estimate that, worldwide, it causes around 140 million cases annually. There are several theories as to why RVVC occurs in immunocompetent patients. One of these includes relapse or reinfection with the same initiating strain [12]. This may be due to failure to clear the original infection due to the formation of antibiotic impermeable biofilms [13] or the development of antifungal resistance [14]. In other patients, it is hypothesized that there may be other factors at play, such as hypersensitivity, resulting in symptoms such as itching in the absence of a curd-like discharge or microbiological confirmation of the overgrowth of candida [15]. However, in the majority of cases, no causative factors can be identified [2].

## 2. The Etiological Agents of VVC

Vaginal candidiasis often presents with pathognomonic symptoms (e.g., curd-like discharge and intense itching) different from those of trichomonas vaginitis (copious green, frothy discharge) and bacterial vaginosis (copious grey, malodourous discharge). The curd-like discharge is attributed to the overgrowth of *Candida* spp. on the vaginal walls, but this overgrowth, in itself, may not be the primary explanation for the intense itching that is also experienced. This is attributed to the inflammatory response and may be unrelated to the number of candida organism present [16]. It is also not currently possible to determine if isolated yeasts are colonizing rather than causing symptoms, as asymptomatic carriage occurs in 15–20% of women [17]. When symptoms do occur, yeast may not be the causative agent [1]. Microscopy demonstrating yeast may aid with diagnosis; however, microscopy is relatively insensitive; thus, women with symptoms of VVC should have a fungal culture performed to confirm diagnosis [18].

In the past, it has been reported that around 85–90% of these infections are attributed to the one species of yeast, *Candida albicans* [19]. However, this may vary with the geographical area (Table 1), the year of study [20] and the study methodology (e.g., isolation methods that do not use chromogenic medium are more likely to miss mixed cultures) [21]. Most studies included in Table 1 describe polyfungal populations, with more than one species of yeast isolated in up to 10% of patients studied. Some studies include patients stratified into those with or without RVVC, whilst in other studies, this is not described (Table 1).

A common theme emerging from the literature and shown by Ratner et al. [22] is that non-*albicans Candida* (NAC) are increasing in frequency and that, since these species have higher rates of fluconazole resistance, they are expected to be seen in many patients with RVVC. As many antifungal medicines are available without a prescription, a lack of adequate antifungal stewardship may result [23]. This lack of antifungal stewardship both in the use of antifungal agents to control human, animal and crop diseases and its use as an anti-fouling agent and in the preservation of timber, is a risk to the further development of resistance in healthcare isolates [24]. Additionally, tolerance to antifungal drugs and persistence has been described for yeast isolated in VVC [25]; thus, routine laboratory testing of antimicrobial resistance fails to detect this, leading to treatment failure. An additional consideration is that the presence of NAC is common in asymptomatic women and that, when considered to be the causative agent of vaginitis, the symptoms may be milder [26].

Isolation of the yeast is important to confirm the clinical diagnosis of candidiasis [8] although it does not distinguish between colonization and causation of symptoms. With the popularity of diagnosis of STIs using molecular tests, some molecular testing platforms also include the detection of yeast DNA. As examples, Navarathna et al. [27] and Lillis et al. [28] included testing for yeast in their molecular vaginitis tests but did not attempt to obtain clinical specificity for candidiasis. Molecular testing could have an important impact in terms of reducing inappropriate therapy for vaginitis, as the turnaround time for results would be significantly reduced. Tse et al. [29] found many empirical treatments were not aligned with the probable causative agent in test results. However, Amerson-Brown [30] notes that the high sensitivity of molecular tests means that many positives will be reported in women who are simply colonized.

Different methods of identification can also yield different descriptions of the etiological agents. The gold standard for identifying yeast is nucleotide sequencing [31]; however, this is expensive and not practical for most diagnostic laboratories. As candida-specific agars are widely used, many laboratories report species based on their appearance on chromogenic agars such as those compared by Scharmann et al. [32]. Caution should be taken with studies that report species-level identification using only chromogenic agar medium. Souza et al. [33] showed that the chromogenic agar they used misidentified *Pichia kudriavzevii* (formerly *Candida krusei*) and reported on two other studies that found similarly. It has also been reported that chromogenic agars may misidentify *Nakaseomyces glabratus* (formerly *Candida glabrata*) [34]. We have found that some batches of chromogenic agar may be inhibitory to low numbers of *N. glabratus* in clinical samples (unpublished data); thus, in a previous trial involving the isolation of yeast from VVC [35], Saboraud’s dextrose agar was used as a second medium to confirm the absence of yeast in samples. The advantage of chromogenic agars for yeast isolation is that they help with the identification of mixed yeast cultures, as the colonial morphology on non-chromogenic medium may be insufficiently different to identify mixtures of species.

In larger laboratories, automated methods for the identification of microbes, such as Vitek 2 systems (which can also perform susceptibility testing) [36,37], and the use of analysers to perform mass spectrometry using MALDI-ToF (matrix-assisted laser desorption ionization combined with time of flight) provide excellent accuracy of speciation of the most commonly isolated yeasts [38].

In a systematic review of VVC that included 39 studies that met the inclusion criteria, the percentage of infections attributed to *C*. *albicans* compared to non-*albicans Candida* (NAC) was found to range from 39% to 88% [39]. To illustrate this, we compiled a summary of reports published within the period of 2021–2025 that used chromogenic agar for isolation of yeast and confirmed their identity using either MALDI-ToF or sequencing to describe the most prevalent species reported in patients with clinically diagnosed VVC (Table 1). Using these selection criteria removed variation inheritably present when less accurate methods are used to isolate and identify causative organisms. It was also noted that no reports from North America, Canada or Australia met these inclusion criteria. The criteria did reduce variation in reported percentages attributable to *C*. *albicans* and NAC.

As NAC are resistant to the most commonly prescribed group of antifungal drugs (the azoles) (reviewed by Gonçalves et al. [40]), it is important to perform microbiological confirmation of the etiological agent of VVC. As an example of the types of resistance that can be seen, in patients attending an antenatal clinic, Aboagye et al. [41] found that less than 50% of the *C*. *albicans* isolates were sensitive to fluconazole and itraconazole. Of concern is that Kroustali et al. [42] reported that 36.5% of their isolates were *Candida parapsilosis*, and recently, it has been reported that this species is becoming increasingly multi-resistant [43]. It has been shown that the availability of over-the-counter antifungal agents has been linked to inappropriate use [44]. Thus, more needs to be done to promote antifungal stewardship to slow the rise of multi-resistant fungal pathogens, although the widespread availability of antifungal agents that do not require prescriptions make this proposal challenging.

A limitation of studies describing the etiological agents of VVC is that isolation of a yeast does not necessary imply that the organism is the causative agent of the symptoms, nor does a negative culture rule out the possibility of VVC. To understand the current limitations of testing, it is necessary to understand host factors such as the immune response and the microenvironment of the vagina.

**Table 1:** Relative proportions of the most common species reported to cause VVC in recent studies where chromogenic agar was used to aid in identification of mixed yeast and using at least MALDI-ToF spectroscopy or sequencing to confirm the identity of isolates (percentages in brackets indicate where RVVC was counted).

Country of Report Number of Women with Culture Positive VVC (% RVVC)	*Candida albicans*	*Candida parapsilosis* Complex	*Nakaseomyces glabratus* Formerly *Candida glabrata*	*Pichia kudriavzevii* Formerly *Candida krusei*	*Candida tropicalis*	Study
Greece 283 *^1^ (5%)	50% (54%)	36.5% (9%)	10% (37%)	3% (0%)	NR	[42]
Portugal *^1^ 61 *^1^ (5.5%)	59%	5%	27%	1.6%	1%	[45]
Brazil *^2^ 101	56%	27%	6%	NR	NR	[46]
Turkiye *^1^ 92 (45%)	61% (68%)	NR	27% (22%)	8% (10%)	2% 0%	[47]
Vietnam *^1^ 237	55%	28%	12%	4%	4%	[48]
Gabon *^1^ 103	62%	7%	15%	9%	5%	[49]
Algeria 29	66%	NR	21%	3%	NR	[50]

Notes: *^1^ Reported to have mixed Candida infections (7–10%). *^2^ Study of military recruits. NR—not reported.

## 3. The Role of Inflammation in the Host Response

### 3.1. Early Steps in Infection

**Figure 1:**
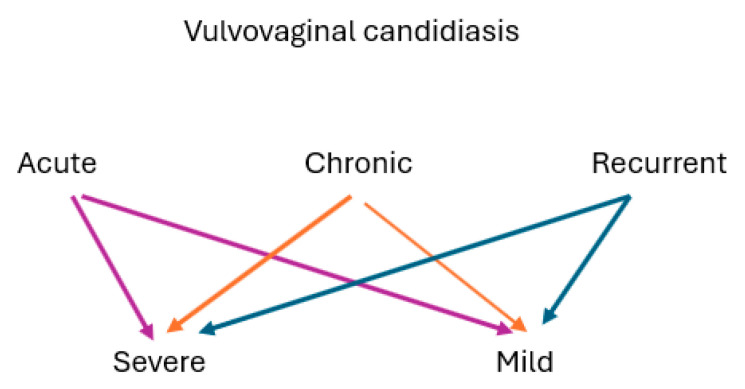
Alternate characterization of the types of VVC.

VVC can manifest in varying degrees of severity, from asymptomatic-to-mild symptoms that resolve spontaneously to more severe forms of the condition that require active management. Sometimes, management is tailored according to the symptoms experienced and the duration of symptomatic episodes [51]. Some authors differentiate between chronic and recurrent VVC because management may differ. Recurrent VVC is generally defined as four or more symptomatic episodes of VVC in a 12-month period [9,10,52]. Chronic VVC may be characterized by prolonged/continuous symptoms [50], while RVVC may be episodic or cyclical [53,54]. Some authors propose that species isolated from women with RVVC demonstrate less virulence than species from those with acute/non-recurrent VVC [55], while other authors consider the severity of symptoms that may occur with both chronic and acute VVC. The severity is thought not to be due to virulence of the yeast but, rather, the host’s inflammatory response to the presence of yeast. It is possible that there is no neat delineation between acute, chronic and recurrent VVC and that overlap may complicate the distinction among these categories. An alternative categorization is demonstrated in Figure 1.

The first step in initiating inflammation on the vaginal walls in the case of *C. albicans* is thought to be the switching from the budding form to a form producing hyphal growth that aids in the anchoring and ingress into the epithelial layer of the yeast cells [56]. This is followed by the expression of numerous adhesins and extracellular proteins that form a matrix; a host immune response is then initiated with the production of cytokines and anti-microbial peptides by epithelial cells [57]. The active form of these pro-inflammatory cytokines needs to be produced through activated cellular inflammasomes and is tightly regulated [58]. This activation is highly inflammatory and leads to cell death, a state called pyroptosis [59]. The sensitivity of the epithelial cell response is thought to be variable enough to enable discrimination between harmless carriage and when an inflammatory response is required due to epithelial cell damage [57]. Moyes et al. [57] commented that the work of Fidel et al. [16] demonstrated, in a human vaginal challenge experiment, that women with RVVC were more sensitive to fungal burdens than those without RVVC.

### 3.2. Internalization by Macrophages and Subsequent Escape

Activation of inflammasomes occurs through the interactions of host-cell receptors with the outer cell-wall components of yeast, which leads to the internalization of the fungus (reviewed by Camilli et al. [58]). When *C. albicans* is internalized by macrophages, it can survive and continue to grow its hyphal forms and destroy the macrophage [60]. This phenomenon is also seen in the two other *Candida* spp. that produce hyphal growth—*C. dublinensis* and *C. tropicalis* [61]—and, to a lesser extent, in *C*. *parasilosis* [62]. Host-cell death is also aided by the production of toxins including candidalysin [63]. For yeasts that escape through hypha-mediated mechanisms, neutrophils attracted to the inflammatory products then attempt to control the fungal infection through the release of neutrophil extracellular traps (NETs) that are composed of neutrophil chromatin and antimicrobial peptides [64]. However, this activity will be impaired if the yeasts are successful in forming a protective biofilm, which is now recognized to be important in yeast survival in RVVC [65].

NAC are also able to survive inside macrophages [66,67] and produce pro-inflammatory cytokines, leading to inflammation [68], and in the case of *N. glabratus*, these cells may enter a dormant state, making them what are called persister cells [69]. This is thought to explain why, in many studies, the proportion of isolations of *N. glabratus* increases in RVVC (Table 1). Although intracellular survival has not yet been demonstrated for *C. parapsilosis*, they have been shown to have the ability to develop tolerance and resistance to fluconazole and form biofilms [43], which may explain the increased isolation for this species (Table 1).

### 3.3. The Influx of Neutrophils

With the failure of macrophages to destroy the yeast, an influx of neutrophils occurs, which is also, in part, mediated by the candidalysin produced in the early stages of hyphal growth [70] and the pro-inflammatory cytokines produced by inflammasomes. Interestingly, the NAC that also produce hyphal growth (*C. dublinensis* and *C. tropicalis*) had less induction of the gene encoding candidalysin [71]. Willems et al. [71] also reported less inflammation in their VVC murine model infected with NAC. The importance of neutrophil recruitment into the vaginal lumen for the major manifestation of inflammation was demonstrated by Fidel et al. [16]. In this seminal experiment of intravaginal inoculation of *C. albicans* into two groups of women (one naïve to VVC and the other, infrequent sufferers of VVC), 15% of women with no history of VVC acquired symptomatic infection, compared to 55% of the group who had previously experienced VVC. They also showed that symptomatic response was associated with an influx of neutrophils and increased yeast burden.

### 3.4. The Role of Adaptive Immunity

The adaptive immune responses that follow the primary innate response were reviewed by Netea et al. [72]. They outline that in this arm of immunity, it is the dendritic cells that signal to T helper 1 cells, which are important for activating macrophage and neutrophil killing of yeast. Richardson et al. [73] reviewed the evidence that memory does occur in the innate immune response, as it has been shown that monocytes exposed to *Candida* can undergo a reprogramming such that they produce increased amounts of inflammatory cytokines on their next exposure.

### 3.5. VVC as an Immunopathology

This failure of both macrophages and neutrophils to kill yeast in the early stages of infection and the resulting inflammation are thought to explain why VVC is considered an immunopathology. This has been reviewed by Yano et al. [74]. Host genetic variation in genes contributing to host defence is thought to explain a small subset of those experiencing RVVC [75]. Diabetes does compromise the host’s ability to control the growth of yeast on mucosal surfaces [76], and with the rising incidence of diabetes, this would be expected to lead to more mucosal infections needing treatment.

The evidence for whether VVC can be thought of as a failure of the adaptive immune response was reviewed by Peters et al. [77]. It is known the patients with cell-mediated immunity (CMI) deficiency are susceptible to mucosal candidiasis [78]. Rosati et al. [75] noted that current evidence fails to clarify whether adaptive immunity plays a significant role in protection from RVVC, as patients with CMI deficiency are no more susceptible to VVC than those who are immunocompetent. Additionally, Wozniak et al. [79] were unable to demonstrate a definitive protective role for antibodies in a mouse vaginitis model.

Significantly, in some women who suffer from vulvovaginitis, very few yeasts are isolated, or it can be difficult to isolate yeast [80]. The first reports suggesting that this symptomatology could be linked to hypersensitivity to *Candida* were published in the 1970s [81,82], followed a report by Witkin et al. [83], who measured elevated IgE in vaginal fluid from some women with this possible hypersensitivity disorder. Bernstein et al. [15] reviewed the evidence for hypersensitivity to *Candida* and reported that women who are treated with immunotherapy show improvement in symptoms, but they concluded that studies are required involving double-blind placebo trials, as well as further studies into the mechanisms of this possible hypersensitivity.

### 3.6. Opportunities to Mitigate Against Immunopathology

It is possible that different microbial members of the vaginal microenvironment can modulate the local immune response. Niu et al. [84] investigated the use of one strain of lactobacilli (*Lactobacillus crispatus* American Type Culture Collection 33820) and found that it could ‘attenuate the virulence of *C. albicans*, modulate the secretion of cytokines and chemokines, and enhance the immune response of VK2/E6E7 cells in vitro’. This study points to important directions for future research to determine whether probiotics and their composition can reduce hypersensitivity in individuals with RVVC.

The response by the host that leads to symptoms in VVC is a localized rather than systemic response [84]. In fact, Fidel et al. [16] showed that in healthy women challenged intra-vaginally with yeast, symptoms were linked with the presence of inflammatory cells rather than their absence. This was corroborated by the work of Giraldo et al. [85].

Efforts to improve the localized vaginal immune response were reported by Ricchi et al. [86]. They used a bacterial lysate to stimulate an in vitro cell culture of human vaginal epithelial cells to increase the production of mitochondrial reactive oxygen species. They also used this lysate to stimulate murine macrophages to improve phagocytic killing of *C*. *albicans*. However, it is unknown whether such stimulation will reduce the recurrence of VVC.

Considering the burden of RVVC being around 8% of women who have experienced VVC, a vaccine to prevent VVC would be welcomed. In the light of the virulence traits of hypha-producing *Candida*, it is hoped that efforts directed to producing neutralizing antibodies to the proteins generated during invasion could lead to protection in some women [87].

Currently, there are no approved fungal vaccines [88]. Several vaccines targeting candidiasis are in development or undergoing trials [89,90,91,92,93,94]. Wychrij et al. [88] reported that their pan-fungal vaccine (based on a peptide conserved in many yeast species), NXT-2, reduced inflammation and provided antibody-mediated protection in a murine model of VVC. Edwards et al. [92] evaluated the effects of administration of their trial vaccine to 178 women with RVVC. They measured B- and T-cell responses after one intramuscular dose. They found immune responses that resulted in a statistically significant number of women experiencing fewer recurrent symptoms and will continue to further develop their vaccine and guidance for administration. Considering that a subset of these women may be experiencing symptoms due to hypersensitivity rather than overt infection with *C*. *albicans*, it will be of great interest to see if a registered vaccine that prevents the initial onset of RVVC is also able to reduce the number of women who go on to develop hypersensitivity. The vaccine designed by Edwards et al. [92] uses a protein from the hyphal tip of *C*. *albicans* to elicit immunity. An alternative immunogen reported by Wychrij et al. [88] aims to protect against a wider range of fungal species, which, at first glance, may seem to hold more promise against the wider range of species reported in RVVC. However, a successful vaccine will reduce the number of women experiencing VVC and, therefore, those going on to develop RVVC, in addition to reducing the pressure towards evolving fungal resistance.

## 4. The Impact of Biofilm Formation in VVC

Microbial cells can exist freely in solution (a state known as planktonic) or in close association with other cells, including those of different species and held together by an extracellular matrix forming what is known as a biofilm. These biofilms help protect the enmeshed microbial cells, including yeast, from the immune system and antimicrobial drugs [95,96].

It is possible that certain strains of bacteria are able to produce molecules that moderate the action of other microbes growing in the surrounding milieu. Hu et al. [97] showed that *Lactobacillus* strains produce metabolites that inhibit the growth of *C*. *albicans*. It has also been shown that *Lactobacillus crispatus* exerts growth inhibition on *C*. *albicans* and that women who have a predominance of *L*. *crispatus* in their vaginal microbiota are less likely to have *C*. *albicans* in their vaginal microbiota [98]. Parolin et al. [99] showed that a variety of *Lactobacillus* species were able to inhibit hyphal formation in yeast and impair biofilm formation. Also, it has been shown that butyrate [100] and allicin from garlic [101] are able to downregulate the production of pseudohyphae in *C*. *albicans*.

In a review of biofilms, Donlan [102] notes that microbial cells in biofilms exhibit altered behaviours, including reduced growth rates and differential gene expression. Some of the biofilm-associated genes have been shown to be expressed at higher levels in isolates from patients with RVVC compared to healthy controls [103]. These reduced growth rates and the presence of a matrix is thought to impair the efficacy of antimicrobial treatment [65]. Biofilm formation has also been shown to help the persistence of candida cells, leading to RVVC [65]. Another physiological state that is produced by *Candida* spp. during biofilm formation is the formation of extracellular vesicles, which contribute to the biofilm matrix. These vesicles contain a variety of peptides, some of which have been shown to be proinflammatory [68,104].

Although many species of bacteria are found within the vaginal microbiota, a study in 2011 found that 95% of the bacteria were lactobacilli [105]. Lactobacilli produce biofilms, and recently, the question of whether lactobacilli could inhibit the growth of *C*. *albicans* was examined by Giordani et al. [106]. They found that biofilm formation by *C*. *albicans* was inhibited in a dose-dependent manner by the exopolysaccharides produced by certain lactobacilli and that the effect was stronger in *Lactobacillus gasseri* than *Lactobacillus crispatus*. Further to this, it is not only the species of lactobacilli that may be important but also the strain. Bae et al. [107] examined 18 strains of *Lactiplantibacillus plantarum* but only selected three based on their ability to inhibit the growth and biofilm formation of *C*. *albicans* and *Gardnerella vaginalis*. Additionally, these three strains also eliminated mature biofilm formation by *C*. *albicans* in their model.

Future studies should address whether the presence of biofilms containing yeast cells can be eradicated. As outlined above, certain *Lactobacilli* species can discourage biofilm formation. Inorganic boric acid has been used in mainstream management of azole-resistant RVVC [108]. Boric acid can interfere with biofilm formation and the development of hyphal transition [109]. However, the elimination of established biofilm is more challenging [110]. What is not known in RVVC is whether specific treatments can help to eradicate established candida-containing biofilms. Wang et al. [110] addressed this challenge by developing an anti-biofilm hydrogel with nanoparticles that enhanced the penetration and delivery of anti-biofilm signalling molecules in a mouse vaginal candidiasis model, resulting in reduced a thickness of biofilm and less inflammation. It will be important to combine these novel therapeutic modalities with probiotics such as the anti-biofilm species mentioned above.

## 5. Clinical Management of VVC

### 5.1. Initial Evaluation

At the outset, it is recommended that mycological evidence of candida colonization be established, although due to the daily fluctuations in vaginal colonization of candida, a negative swab result does not always rule out RVVC [111]. Ruling out other conditions such as sexually transmissible infections or skin conditions causing itching, such as lichen sclerosis, is also important.

### 5.2. Management of RVVC

Mainstream management of RVVC usually consists of suppressive and maintenance treatment with oral or topical antifungals (see Table 2), following mycological confirmation on at least one occasion [2]. This is the only method of management supported by a multicentre randomized controlled trial [52] and remains the most widely recommended method of management according to national and international guidelines [54,112,113,114]. However, advising women that this will cure them could be unrealistic, as 50–80% of women undergoing treatment for RVVC will experience recurrences after the maintenance therapy has ceased [3,115].

### 5.3. Antifungal Agents Used in VVC

A number of oral and topical azoles are used in the management of vulvovaginal candidiasis. Azoles consist of imidazoles (e.g., clotrimazole, miconazole and butoconazole), triazoles (e.g., fluconazole, itraconazole and voriconazole) and tetrazoles (oseconazole) [116]. Table 2 lists the azoles most recommended in some clinical guidelines, but other azoles may also be used.

The use of boric acid in suppression and maintenance therapy has not been evaluated in randomized controlled trials for recurrent vulvovaginal candidiasis. Canadian guidelines are the only guidelines currently recommending boric acid in suppression and maintenance therapy [117]. It has been documented for use in non-albicans infections; however, there are concerns regarding its impact on the vaginal microbiota. It is often available in compounding pharmacies and is not considered safe during pregnancy.

Fluconazole, a triazole, is most commonly used for suppression and maintenance therapy due to its tolerability, accessibility and cost [118]. Gupta et al. [119] outlined the various side effects and drug interactions associated with fluconazole. Side effects such as headache, nausea, stomach pain and rash are generally mild and occur in less than 1:100 people. However, rarely, hepatotoxicity can occur in patients with underlying liver conditions, and liver function tests are recommended for this group. Other disease interactions include QT prolongation, usually in patients with multiple comorbidities. Fluconazole can also interact with other medications to potentially cause serious side- effects, and care (increased monitoring or dose adjustment) is needed when prescribing fluconazole long-term. Potentially harmful interactions include blood thinner warfarin, some statins, amiodarone, common anti-platelet medication clopidogrel, phenytoin and antidepressants such as citalopram.

Older azoles such as itraconazole [120] are not usually recommended as first-line treatment for RVVC due to the need for more frequent dosing and the comparative efficacy of fluconazole.

Donders et al. [51] reported that individualized, reducing doses of fluconazole if symptom-free over a 12-month period may prevent clinical relapses for women experiencing recurrent vulvovaginal candidiasis. In this study, 117 women started with a loading dose of 600 mg of fluconazole in the first week, then, for two months, took 200 mg twice weekly, followed by 200 mg every two weeks for four months, then 200 mg monthly for six months, according to their individual response. Of the women that commenced the trial and achieved mycological and symptom cure after induction, 90% were disease-free after six months, and 77% were disease-free after 12 months (however, only 36 completed the full treatment protocol and the final follow-up visit). Of note with respect to this study is the variety of responses and fluconazole doses needed to eliminate symptoms and mycological evidence of candida (women were categorized into optimal, sub-optimal and poor responders).

There is no consensus about whether oral or topical therapy is superior in efficacy. One systematic review suggested no difference in the efficacy of oral versus topical anti-fungal treatment in VVC [121], and a more recent systematic review concluded that neither method demonstrated superiority in terms of reduced recurrences at six months [122].

Expert consensus concludes that there is rising evidence that women on long-term suppressive therapy may be susceptible to developing resistance to future suppressive regimens [115]; hence, change of therapy or increasing the strength of therapy may be indicated, although new therapeutic options are urgently needed [123].

### 5.4. New Drugs on the Horizon

A novel tetrazole with a side-effect profile similar to that of fluconazole, oteseconazole, has been approved for use in the USA, as well as ibrexafungerp, a triterpenoid-glucan synthase inhibitor, which may be effective against isolates resistant to fluconazole [124], but approval in other countries is pending. Oteseconazole has a long elimination half-life of 138 days [116] and was found to be 40 times more effective against most *Candida* spp. It also showed promise in clinical trials against fluconazole-resistant *C. albicans* [125]. These therapies have not been included in the most recent clinical practice guidelines, and regimens vary.

New topical treatments are the subject of clinical trials [126]. Sertaconazole shows similar efficacy to clotrimazole [127]. Olorofim, an orotomide (primarily for *Aspergillus* infections), and fosmanogepix (a Gwt-1 inhibitor that may be useful for azole resistance) are expected to be approved by the FDA in the coming years [125]. Other novel antifungals are also in phase 2 and 3 clinical trials but are primarily used for systemic fungal infections in immunocompromised patients [125].

### 5.5. Management of VVC in Challenging Conditions

Some conditions pose additional challenges in the management of RVVC, as some therapies are not recommended in certain situations and, sometimes, mainstream management may be ineffective.

- Pregnancy

Pregnancy hormones (particularly estrogen) can cause vaginal epithelial cells to become more susceptible to the adherence of candida. Increased vaginal secretion of glycogen can also promote the growth of fungal hyphae [128]. Topical rather than oral azoles are recommended in RVVC during pregnancy due to possible teratogenic effects of fluconazole [129,130], although some sources dispute this association [131]. Boric acid pessaries are not recommended. Polyenes such as nystatin may also be used but are less popular due to their more frequent administration. Oral probiotics have not been shown to reduce recurrences of VVC in pregnancy in women with RVVC [132]. There may be a place for amphotericin lozenges inserted vaginally. Some studies have shown promise [133,134], although such interventions have yet to be evaluated in large clinical trials.

b. Diabetes

If diabetes is uncontrolled, increased vaginal glycogen can promote the adhesion of candida in vaginal tissue [128]. Most management guidelines suggest that a longer period of treatment may be recommended for patients with diabetes, but few recommend specific management strategies for poor responders, apart from checking for co-morbid dermatological conditions such as lichen sclerosis [118]. When blood sugar levels are well controlled in diabetic patients, they are less likely to experience flare-ups during anti-fungal therapy. However, certain medications for diabetes that prevent glucose reabsorption in the proximal tubule, such as SGLT2 inhibitors, can result in glucosuria, which can exacerbate VVC [9,118].

c. Immunocompromise

Immunocompromised or critically ill patients are more likely to experience invasive candidiasis than healthy women, who are more susceptible to mucocutaneous infection such as vulvovaginal candidiasis, as the compromised immune system allows overgrowth of Candida [135]. In women living with HIV, rates of VVC were found to be higher when CD4 and T-cell counts were less than 200 cells/mm3 and the viral load of HIV RNA was more than 10,000 copies/mL [136]. Some studies have found resistance to fluconazole in women with HIV and have recommended polyenes such as nystatin or amphotericin for management in this population [137].

More intensive therapy is usually recommended for immunosuppressed patients [128]. This may include higher doses or more frequent administration of antifungal agents.

d. Resistance to fluconazole and other azoles

Although fluconazole-resistant *C. albicans* (FRCA) accounts for less than 5% of isolates from women with RVVC [115], there are growing concerns about women with fluconazole and other azole resistance to both *albicans* and *non-albicans Candida* [8]. Cross-resistance to other azoles is also common with FRCA [123]. According to Li et al. [20], *Candida albicans* is the dominant pathogen associated with anti-fungal resistance; however, *Candida glabrata* resistance is on the rise. With suspected resistance, identification of species is recommended to guide management [135]. Alternatives to fluconazole include nystatin, boric acid, terconazole and amphotericin B vaginal pessaries or cream [116]. Itraconazole may be effective against many clinical isolates of *C. parapsilosis, N. glabratus* and *P. kudriavzevii* that are resistant to fluconazole but is less well tolerated [116].

e. Co-existing infection with bacterial vaginosis

In some populations, bacterial vaginosis (BV) and VVC may be linked, although the aetiology of this is yet to be confirmed [52]. Since bacterial vaginosis (BV) is usually managed with antibiotics, women with concurrent BV and RVVC face management challenges, as antibiotics are considered a predisposing factor for VVC [138]. Alternative management strategies could include boric acid pessaries, which penetrate the biofilm [109]; however, their efficacy has yet to be proven in randomized controlled trials. Early in-vitro studies with a biological bacteriostatic agent showed promise, but such interventions have yet to be evaluated in randomized controlled trials [139].

### 5.6. Complementary and Alternative Therapies

Generally, the evidence for the use of complementary and alternative medicine (CAM) in genital candidiasis is not strong. The use of CAM in most countries is not regulated, nor is government approval required, and these substances are not subject to the same rigorous research and trial processes required for mainstream medicine. Consequently, there is no guarantee of the product’s composition or formula. There is the possibility that some CAM products may interfere or interact with mainstream treatments. However, anecdotally, many women report improvement of symptoms following use. Some clinical trials have shown the promise of CAM interventions such as propolis [140], *Ageratina pichinchensis* extract [141] and *Allium sativum* (garlic) [142]. Other clinical trials have found that, despite in vitro efficacy, some natural products do not provide long-term relief for VVC. Sardi et al. [128] proposed that the reason for this is that many of the biologically active constituents of these therapies are ineffectively absorbed. These constituents include flavonoids, tannins and terpenes.

Natural therapies include but are not restricted to dietary changes including probiotics and yoghurt, herbal supplements such as pau d’Arco, and *Melaleuca alternifolia* (tea-tree oil (which can result in irritation, burning and redness). Excess use of other alternative therapies may have systemic effects (e.g., bleeding with garlic) [143]. Where a woman finds relief with a particular alternative or complementary medicine, if it causes no harm, it should not be discouraged, as management should be individualized, whether mainstream or alternative.

Evidence for treatment of sexual partners is not currently well supported by data [122], although if male partners have symptoms of candidiasis, topical antifungals are often recommended [112].

## 6. The Influence of the Vaginal Microbiota on Candidiasis Outcomes

### 6.1. Intestinal Carriage of Candida spp.

The intestinal tract is probably the source from which colonization of the vagina occurs initially [144]. Thus, it is worth considering whether efforts to lower intestinal carriage could prevent VVC. *C*. *albicans* was reported in the intestinal tract of 83% of 695 healthy volunteers by Delavy et al. [145], who investigated the factors influencing the intestinal growth of *C*. *albicans*. They found that these factors were multifactorial whilst noting that eating between meals and a low-sodium diet was linked to higher intestinal carriage. Of interest was the finding that other members of the host microbiota might be associated with lowered numbers of *C*. *albicans* in the intestine. They speculated whether this might be due to higher amounts of short-chain fatty acids (SCFAs) produced in these individuals. The positive influence of SCFAs to produce beneficial outcomes in terms of *C*. *albicans* colonization was reviewed by McCrory et al. [146], who noted the inhibition of *C. albicans* growth and switching to the invasive hyphal form, as well as downregulation of the surface molecules of yeast that communicate with immune cells. Nguyen et al. [100] also reported that SCFA butyrate (which is produced by intestinal bacteria) reduced biofilm formation and enhanced killing by fluconazole in both *C. albicans* and *C. parapsilosis*. It also increased the macrophage phagocytosis rate and killing of internalised *C*. *albicans*.

The role of a high-fibre diet in vaginal health may provide important insights, as the SCFAs produced in high-fibre diets by intestinal bacteria may control the levels of yeast colonization and the behaviour of yeast. As more people realize the importance of resuming a high-fibre diet, which has been lost in Western diets [147], it will be possible to perform large studies comparing the vaginal health histories of those with and without a high-fibre diet. There is evidence that a diet high in animal protein derived from red and processed meat, as well as alcohol consumption, is associated with less a protective vaginal microbiota [148]. Further microbiome studies of the vagina, coupled with vaginal health history and probiotic interventions, should inform the rational use of probiotics.

### 6.2. The Importance of Community State Types

In healthy pre-menopausal women, the most predominant member of the local vaginal microbiota is *Lactobacillus* spp. [105]. Ravel et al. [105] reported that the vaginal microbiomes of 396 asymptomatic women could be characterized into five Community State Type (CST) groups, of which four were dominated by different *Lactobacillus* species: Group I, *L*. *crispatus* (26.2% of women); Group II, *L*. *gasseri* (6.3%); Group III, *L*. *iners* (34.1%); and Group V, *L*. *jensenii* (5.3%). Group IV had lower numbers of lactobacilli and a higher proportion of anaerobic species and was found in 27% of women. Some similarities in groupings were reported a year later by Smith et al. [149]; however, only 10 women were included in their study. Kim et al. [150] reported on the CST groups of 40 women with recurrent vaginitis (aetiology not determined) and found that 21 (52.5%) were classified as CST IV, eight (20.0%) as CST III, 5 (12.5%) as CST I, two (5.0%) as CST II, one (2.5%) as CST V and three (7.5%) as mixed CST, confirming the association of a shift in *Lactobacillus* species predominance in cases of recurrent vaginitis.

### 6.3. The Use of Probiotics to Improve Vaginal Health

Following on from this, one would expect positive outcomes for the use of oral probiotic supplementation or vaginal administration. However, studies of vaginal probiotic use should be scrutinized for choice of *Lactobacillus* spp. As an example, Jepson et al. [151] published a study refuting that the use of probiotics has a role in fertility outcomes. In their literature search, they noted that Koedooder et al. [152] reported that the presence of *L*. *crispatus* was associated with greater fertility outcomes, yet they elected to use the less common *L*. *gasseri* species in their study. Perez et al. [153] reported that their strain of *L*. *gasseri* was superior to *L*. *crispatus* in its colonization ability. France et al. [154] reviewed aspects of *L*. *crispatus,* concluding that it makes a good candidate for probiotic use.

Tortelli et al. [98] showed that in women whose lactobacilli were dominated by *L*. *iners*, there was a significant association with the presence of *C*. *albicans*, whereas, conversely, women predominantly colonized with *L*. *crispatus* had a significantly lowered association with *C. albicans*. They also found that in vitro, the cell-free supernatant of *L*. *crispatus* slowed the growth of *C*. *albicans*. This is interesting, as *L*. *iners* has been associated with disease states; for, example van Houdt et al. [155] showed that those with a vaginal composition predominated by *L*. *iners* were significantly more likely to be infected with chlamydia over a one-year period. Of additional interest is the finding by McKloud et al. [103] that an in vitro experiment where *L. crispatus* was co-incubated with a polymicrobial biofilm containing *C. albicans* resulted in decreased fungal burden in the biofilm over a 48 h period.

What is less clear, however, is whether the presence of particular species or strains of lactobacilli exert an influence over whether symptoms will occur when *Candida* spp. grow and the way in which these species grow (e.g., switching to hyphal forms and expression of biofilm extracellular components). Evidence is beginning to emerge that it is not only the species of lactobacilli that is important but also the strain. As an example, Armstrong et al. [156] reported that *L*. *crispatus* strain CTV-05 was able to reduce the recurrence of bacterial vaginosis. However, colonization by this strain was lost in half the women after three months [157].

Thus, assertions that because Lactobacilli are predominant in so many women with VVC, they must not be providing a protective effect need to be questioned, as in the past, we did not have the opportunity to provide metagenomic analysis of the microbiome, which reveals specific functional characteristics of particular strains. It will be important in the future to perform these metagenomic studies on those who have not experienced VVC or who have recovered quickly from VVC and compare these findings to those for patients who suffer from RVVC.

Moreover, as some women with RVVC experience symptoms due to hypersensitivity (described in Section 3), the following question arises: ‘Are some species of lactobacilli able to provide beneficial host immunomodulated responses?’ A reduction in inflammation is seen in women who have a vaginal microbiota dominated by *L*. *crispatus* [158]. Gosmann et al. [158] also postulated that this reduced inflammation could explain why women with an *L*. *crispatus*-dominated vaginal microbiota had less infection with HIV due to the lowered presence of HIV’s target CD4 T cells. The potential for lowered inflammation and prevention of RVVC in women whose microbiota is predominantly *L*. *crispatus* should be explored. This should be followed by research to show whether lasting colonization of *L*. *crispatus* can be effected in women with a microbiota predisposing to inflammation.

### 6.4. Efforts to Manipulate the Local Vaginal Microbiota

Transplantation of microbiota from healthy donors has been used to ameliorate disease symptoms of the gut [159] and to improve health markers of obesity [160]. Recently, vaginal microbiome transplants (VMTs) were performed to treat intractable bacterial vaginosis in five women [161], with repeated administration required in three and failure to resolve symptoms in one recipient. Building on these findings, Wrønding et al. [162] showed proof of concept that donor material could be screened, stored and administered with lasting effects in a patient with repeated pregnancy loss and associated vaginal symptoms of bacterial vaginosis. The predominant species in the donor material was *L. crispatus*. Future studies should report on whether VMT in women with intractable RVVC leads to a lasting repopulation of *L*. *crispatus* and resolution of RVVC. Preparation of transplant material is onerous and not without health risks, so should only be performed for research purposes until sufficient evidence leads to regulatory approval.

Current interest has been focused on the use of *L. crispatus* containing probiotics to ameliorate conditions associated with genitourinary health. Liu et al. [163] reported that intravaginal administration of a healthy donor-derived strain of *L. crispatus* (strain chen-01) was able to establish and reduce the viral shedding load in human papilloma virus (HPV)-infected women. Their work followed the finding that women with cervical cancer were less likely to have *L*. *crispatus* predominating in their vaginal microbiota [164]. Hemmerling et al. [165] reported that intravaginal administration of *L*. *crispatus* was able to lower inflammation and shift the microbiota to being lactobacillus-predominant in a population of women at risk of HIV infection. El-Baz et al. [166] performed in vivo experiments to lower group-B streptococcal (GBS) colonization, with the ultimate aim of effecting less GBS colonization of pregnant women and lowering the need for intra-partum administration of antibiotics. Ideally, future studies will establish if *L*. *crispatus* can be administered daily with food such as yogurt to effect long-term colonization. An alternative approach has been proposed by Ravel et al. [167], who combined three strains of *L*. *crispatus* to produce a multi-strain consortium with proven ability to inhibit growth of *Gardenerella* spp. and *Candida* spp. *(C. albicans*, *C*. *tropicalis* and *C*. *parapsilosis*). The formulation of the vaginal tablet also included substates to encourage the growth of the microbial cargo in a slow-release manner. In their trial, they found a significant reduction in *Candida* spp. in the trial participants.

## 7. Conclusions

Broad principles of management can be applied with RVVC, as the evidence suggests that there is no ‘one size fits all’ approach that will cover every individual who experiences this condition. Management will be dictated by personal preference and tolerance of therapy (oral versus topical; mainstream versus alternative and complementary), individual host responses to candida, co-existing diseases or health conditions, the availability of antifungal treatment, immune factors and a complex interplay of the vaginal microbiota.

RVVC is a significant problem that is complicated by rising resistance to antifungal agents. In current guidelines, the recommended first-line management of this condition is prolonged antifungal treatment. Alternative considerations include a more nuanced approach to the treatment and monitoring of RVVC. This could involve describing a patient’s vaginal microbiome by sequencing and reporting the present community state types, although this approach is circumvented by widespread self-management due to the ready availability of non-prescription antifungals, as well as challenges with budgetary constraints.

Current research indicates that *Lactobacillus crispatus* can exert beneficial control over other organisms present in the microenvironment and reduce inflammation. Based on personalized descriptions of the vaginal microbiome, targeted probiotics could be selected and administered. Ultimately, it is hoped that an effective vaccine may help to reduce the incidence of RVVC; however, there are still many challenges associated with its development, as the strains of yeast causing RVVC are varied and ubiquitous. However, opportunities abound for research to continue to explore the various strains of beneficial resident vaginal lactobacilli, their administration and whether personalized medicine can move to a preventative phase where candida-containing biofilms can be prevented rather than allowed to persist and promote inflammation. Finally, long-overdue insights into vaginal ecology are beginning to be described as we move forward into a more positive era of the promotion of vaginal health.

## Figures and Tables

**Table 2:** Antifungal agents and recommended dosing.

Suppression	Dose	Comments	Source
***Oral azole***			
Fluconazole	150–200 mg every 3–5 days for 3 doses/up to 2 weeks.	May need private script Oral. Not suitable during pregnancy or breastfeeding	[54] [112] [115]
***Topical imidazoles such as:***			
Clotrimazole	10% vaginal cream nocte for 10–14 nights OR 500 mg every 3–5 days for up to 2 weeks vaginally	Vaginal pessaries or cream. Suitable during pregnancy	[113] [115] [116]
Miconazole	1200 mg every 3–5 days for up to 2 weeks	Vaginal suppository	[115] [116]
Terconazole	0.8% 5 g/day for 3 days 80 mg/day for 3 days	Vaginal cream Vaginal pessary	[115] [116]
***Polyene***			
Nystatin	100,000 IU inserted vaginally for 14 days	Vaginal cream Vaginal pessary	[55] [115]
**Maintenance**	**Dose**	**Comments**	
***Oral azole***			
Fluconazole	150–200 weekly. Oral	Oral. May need private script Not suitable during pregnancy or breastfeeding	[2] [54] [116] [51] [112] [115]
***Topical imidazoles such as:***			
Clotrimazole	500 mg every week	Intravaginal pessaries or cream Suitable during pregnancy	[115] [116]
Miconazole	500 mg every week	Vaginal suppository Suitable during pregnancy	[115] [116]
***Polyene***			
Nystatin	100,000 IU inserted vaginally 2–3 times/week for 6 months	Vaginal suppository Suitable during pregnancy	[115] [116]
***Other***			
**The length of recommended maintenance therapy varies with different guidelines but is generally 6–12 months.**			

## Data Availability

No new data were created or analyzed in this study. Data sharing is not applicable to this article.

## Abbreviations

The following abbreviations are used in this manuscript:

BV	Bacterial vaginosis
CAM	Complementary and alternative medicine
CST	Community state type
GBS	Group-B streptococcal
GP	General practitioner
NAC	Non-*albicans Candida*
SCFA	Short-chain fatty acid
RVVC	Recurrent vulvovaginal candidiasis
VVC	Vulvovaginal candidiasis
